# Asthma Exacerbations Following Wildfire Smoke PM_2.5_ Exposure Among U.S. Adults

**DOI:** 10.1029/2026GH001972

**Published:** 2026-11-07

**Authors:** Yanhong Huang, Li Luo, Shuguang Leng, Su Zhang, Roberta P. Lavin, Michaela Buenemann, Yan Lin, Matthew Campen, Xi Gong

**Affiliations:** ^1^ Department of Veterinary Physiology and Pharmacology Texas A&M University College Station TX USA; ^2^ Department of Biobehavioral Health The Pennsylvania State University University Park PA USA; ^3^ Department of Geography & Environmental Studies University of New Mexico Albuquerque NM USA; ^4^ Department of Internal Medicine University of New Mexico Albuquerque NM USA; ^5^ University of New Mexico Comprehensive Cancer Center Albuquerque NM USA; ^6^ Lung Cancer Program Lovelace Biomedical Research Institute Albuquerque NM USA; ^7^ Earth Data Analysis Center (EDAC) University of New Mexico Albuquerque NM USA; ^8^ Center for Health Equity and Preparedness College of Nursing University of New Mexico Albuquerque NM USA; ^9^ Department of Geography and Environmental Studies New Mexico State University Las Cruces NM USA; ^10^ Department of Geography The Pennsylvania State University University Park PA USA; ^11^ Social Science Research Institute (SSRI) The Pennsylvania State University University Park PA USA; ^12^ Department of Pharmaceutical Sciences College of Pharmacy University of New Mexico Albuquerque NM USA; ^13^ Institute for Computational and Data Sciences (ICDS) The Pennsylvania State University University Park PA USA

**Keywords:** wildfire smoke PM_2.5_, asthma exacerbation, case‐crossover study, *All of Us* research program, environmental health

## Abstract

Asthma exacerbation can be triggered by various forms of air pollution, including PM_2.5_ from wildfire smoke. This study aims to investigate the association between wildfire smoke PM_2.5_ exposure and asthma exacerbations in adults during the warm months (April–August) between 2006 and 2019. Using a case‐crossover design, we analyzed 18,935 asthma exacerbation events and four matched control periods per event from the National Institutes of Health (NIH) *All of Us* research program. We used the conditional logistic regression to estimate the association (odds ratios [ORs]) between wildfire smoke PM_2.5_ exposure and asthma exacerbation, considering lag periods of 0–7 days and adjusting for temperature, precipitation, and humidity. Significant positive associations were observed across all lag periods, with adjusted ORs ranging from 1.27 (95% CI: 1.22–1.33) to 1.38 (95% CI: 1.32–1.44). Stratified analyses showed higher odds of asthma exacerbation among older adults, non‐Hispanic Whites, midwestern residents, and those exposed in May. These findings highlight the potential contribution of wildfire smoke PM_2.5_ to asthma exacerbations in adults and the importance of targeted public health strategies to reduce exposure during wildfire events.

## Plain Language Summary

Wildfire smoke is becoming more common in many parts of the United States and contains fine particles (PM_2.5_) that can harm human health. This study examines how exposure to wildfire smoke affects asthma exacerbations in adults. We analyzed nearly 19,000 asthma exacerbation events from the *All of Us* research program between 2006 and 2019 during the warm season (April to August). For each event, we compared pollution levels shortly before the asthma attack with other control periods for the same individual. We found that higher exposure to wildfire smoke PM_2.5_ was consistently associated with an increased risk of asthma exacerbations, even several days after exposure. The risk was higher among older adults, non‐Hispanic White individuals, people living in the midwestern United States, and during the month of May. These findings suggest that wildfire smoke has important short‐term impacts on respiratory health and highlight the need for improved air quality warnings and public health strategies to reduce exposure, especially for vulnerable populations.

## Key Points

- Wildfire smoke PM_2.5_ exposure is associated with increased risk of asthma exacerbations in adults across multiple lag days
- Stronger associations were observed among older adults, non‐Hispanic Whites, the midwestern regions, and during May
- Findings highlight the need for improved public health strategies to reduce wildfire smoke exposure and asthma risk

## 1 Introduction

Asthma is a chronic lung disease that affects people of all ages (WHO, 2025). It is caused by inflammation and muscle tightening around the airways, which makes it harder to breathe. In 2019, asthma affected an estimated 262 million people worldwide and was responsible for approximately 455,000 deaths (WHO, 2025). In the United States of America (USA), between 2001 and 2021, the age‐adjusted asthma death rate was 12 per million people, with adults experiencing a mortality rate over six times higher than that of children (U.S. CDC, 2023b). Additionally, asthma prevalence was higher among females than males, while non‐Hispanic White individuals exhibited the lowest prevalence compared to other racial/ethnic groups in the USA during the same period (U.S. CDC, 2023a).

Frequent and severe asthma exacerbations are a hallmark of severe asthma, yet the pathophysiological mechanisms driving these events remain inadequately understood, thereby impeding the development of effective therapeutic interventions (Wenzel, 2011). Asthma exacerbations typically occur when symptoms acutely worsen in response to external triggers such as air pollution, allergens, respiratory infections, or psychological stress (Ritz et al., 2016; U.S. CDC, 2024). Extensive epidemiologic evidence links ambient air pollution to higher risks of asthma‐related ED visits and hospital admissions, exemplified by analyses from Barcelona (2008–2016) showing that higher NO_2_ concentrations were significantly associated with increased asthma ED visits and exacerbation‐related hospitalizations (Martínez‐Rivera et al., 2019). Similarly, in the Philadelphia metropolitan region (USA) during 2011–2014, short‐term (within a week) exposure to PM_2.5_ was linked to significantly increased odds of asthma exacerbation in both warm and cold seasons (Huang et al., 2021). Short‐term exposure to ambient air pollutants, including PM_10‐2.5_, PM_2.5_, O_3_, and NO_2_, has been associated with increased odds of asthma‐related emergency department visits, as shown in studies conducted in Seoul, South Korea (2008–2011) and Australia (2013–2015) (Cheng et al., 2022; Kim et al., 2015). Collectively, the evidence highlights the critical role of air pollution in triggering asthma exacerbations, with observed effect heterogeneity attributable to differences in pollutant types, regional environmental contexts, and temporal (seasonal) conditions.

The frequency and intensity of wildfires have increased in recent decades. Globally, the average duration of the fire season rose by 18.7% between 1979 and 2022. In the USA, the number of wildfires has quadrupled since 1983, reaching 64,897 incidents in 2024, while the total area burned has increased more than sixfold (Akdis & Nadeau, 2022; NIFC, 2025). Wildfire smoke is comprised of a complex mixture of particulate matter (e.g., PM_2.5,_ PM_10_), gaseous pollutants (e.g., carbon monoxide), hazardous air pollutants (HAPs, e.g., polycyclic aromatic hydrocarbons (PAHs)), and heavy metals, which collectively impair air quality in the USA (US EPA, 2026). Exposure to wildfire smoke is associated with premature mortality in the general population and has been shown to cause and exacerbate diseases affecting the lungs, cardiovascular system, nervous system, skin, gastrointestinal tract, kidneys, eyes, nasal passages, and liver (WHO, 2024).

A growing body of evidence indicates that wildfire smoke and wildfire‐derived PM_2.5_ are associated with respiratory morbidity, including asthma symptoms, emergency department visits, and hospitalizations in different regions of the world (Al Saadi, 2010; Gan et al., 2020; Liu et al., 2013; Raju et al., 2020; Siddique et al., 2011). However, current research on wildfire smoke PM_2.5_ and asthma exacerbations largely remains limited to short‐term periods or localized regional analyses, highlighting a significant gap in long‐term, nationwide investigations (Aguilera et al., 2021; Stowell et al., 2019; Sugrue et al., 2026; Youssouf et al., 2014; Zhang et al., 2025). To address this knowledge gap, we analyzed the association between wildfire smoke PM_2.5_ exposure and asthma exacerbations among adults across the contiguous USA during the warm months (April–August) from 2006 to 2019. This study focused exclusively on the warm months to align with the peak wildfire season and to minimize confounding from non‐wildfire seasonal respiratory factors (NIFC, 2016). We selected a consistent April‐August window across all regions to preserve the matched case‐crossover structure and to reduce confounding from respiratory triggers that become more prominent in fall and winter, such as respiratory infections. By leveraging a comprehensive and long‐term data set, this analysis aims to provide an in‐depth assessment of temporal, spatial, and demographic variations in asthma exacerbation risks attributable to wildfire smoke PM_2.5_ exposure across the contiguous USA.

This study design allows us to address several questions that prior wildfire smoke and asthma studies could not fully evaluate. By linking a national adult cohort with daily wildfire‐specific PM_2.5_ estimates we were able to examine asthma exacerbation risk across a long study period and across the contiguous USA rather than within a single state, region, or wildfire event. This national scope allowed us to assess whether associations differed by geographic region, demographic characteristics, month, and exposure lag period. In addition, the use of wildfire‐attributable PM_2.5_ estimates, rather than total ambient PM_2.5_ or broad air quality categories, helped reduce exposure misclassification from non‐wildfire pollution sources and provided a more specific estimate of the health risks associated with wildfire smoke.

## 2 Data and Methods

### 2.1 Asthma Exacerbation Data

The adult asthma exacerbation data used in this study were collected from the *All of Us* Research Program (*All of Us*) (NIH, 2025), a nationwide NIH initiative launched in 2018 to build a large, diverse health database for precision medicine research. The program recruits participants from across the United States through healthcare provider organizations and community‐based enrollment, with a particular emphasis on including individuals from diverse and historically underrepresented populations to enhance demographic and geographic representation. *All of Us* collects data from over 1 million participants through electronic health records (EHRs), surveys, physical measurements, and biospecimens to support studies across a wide range of health conditions (NIH, 2025).

In this study, adult asthma exacerbation cases were identified using the *All of Us* Controlled Tier data set (version 7) by extracting all EHR entries corresponding to conditions related to asthma exacerbation (Table S8 in Supporting Information S1) during the warm months (April–August) from 2006 to 2019. To minimize the influence of prior asthma exacerbations and ensure the independence of observations, only the first recorded asthma exacerbation per individual within any 30‐day period was included in the analysis. After applying this criterion, a total of 18,935 asthma exacerbation events from 6,154 adults were extracted as cases. This study utilized a case‐crossover design, with each asthma exacerbation case matched with four control periods for the same individual, occurring on the same day of the week exactly one to 4 weeks prior to the case date (e.g., if the exacerbation occurred on a Monday, the control days were the Mondays one, two, three, and 4 weeks earlier). This approach resulted in a comprehensive data set for assessing the association of wildfire smoke PM_2.5_ exposure and asthma exacerbations. The data set contains detailed demographic and administrative information for each record, including residential location (three‐digit ZIP code), date of birth, condition start date (the date when the instance of the condition is recorded), race/ethnicity, and sex, which allows for a comprehensive investigation of potential disparities and contributing factors.

### 2.2 Wildfire Smoke PM_2.5_ Data

This study used daily estimates of ambient wildfire smoke PM_2.5_ concentrations at the local level across the contiguous USA from 2006 to 2019, developed by Childs et al. (2022). This data set was generated using a machine‐learning approach that integrated ground‐based monitoring, satellite‐derived smoke plume data, air trajectory modeling, and meteorological inputs to isolate wildfire‐attributable PM_2.5_ from other pollution sources. The daily wildfire smoke PM_2.5_ estimates have a spatial resolution of 10 km × 10 km. For this study, these estimates were spatially aggregated to the three‐digit ZIP code level by averaging the concentrations of all 10‐km grid cells whose centroids fall within each ZIP code boundary. This approach produced representative exposure values that capture regional variations while reducing spatial noise from finer‐scale fluctuations. Wildfire smoke PM_2.5_ exposure was assigned at the daily level based on residential three‐digit ZIP code. For the primary analysis, we defined a binary exposure indicator whereby days with any detectable wildfire smoke PM_2.5_ concentration (>0 μg/m^3^) were classified as “exposed,” and days with no wildfire smoke PM_2.5_ (0 μg/m^3^) were classified as “unexposed.”

### 2.3 Meteorological Data

To account for potential confounding by weather and climate factors known to influence respiratory health, we included key meteorological variables into the analysis (Mirabelli et al., 2016; Pini et al., 2022). Daily meteorological data across the USA during 2006–2019, including mean daily temperature (K), mean daily surface precipitation amount (kg/m^2^), and mean daily relative humidity (%), were obtained from the NOAA's National Center for Environmental Prediction (NCEP) North American Regional Reanalysis (NARR) data set. This data set provides detailed atmospheric and land‐surface information for North America at a 32 km by 32 km grid, with data available at three‐hour, daily, and monthly intervals. By assimilating land surface, satellite, and upper‐air observations through a robust data assimilation system, NARR offers a reliable source of climate and weather data widely used in environmental research (NARR, 2011). For this study, the meteorological data were also aggregated to the three‐digit ZIP code level by calculating daily averages, thereby aligning the data spatially and temporally with health outcome records across the contiguous USA.

### 2.4 Statistical Analysis

This study utilized a case‐crossover design with conditional logistic regression to assess the association between wildfire smoke PM_2.5_ exposure and asthma exacerbations in adults during the warm months (April–August) from 2006 to 2019. Each case served as its own control, with control days matched to the same day of the week within the four weeks prior. This design inherently controls for seasonality, day‐of‐week effects, and time‐invariant individual characteristics such as race/ethnicity, age, and sex over the short term, thereby minimizing potential confounding. Wildfire smoke PM_2.5_ exposure was assigned to each case and its matched control periods using exposure data aggregated at the three‐digit ZIP code level for the corresponding dates. The odds ratios (ORs) were calculated to quantify the association between wildfire smoke PM_2.5_ exposure (binary, exposed or unexposed) and asthma exacerbation. We evaluated wildfire smoke PM_2.5_ exposure from 0 to 7 days prior to the case or control date (lags of 0–7 days) to assess potential delayed effects on asthma exacerbation. The model was adjusted for potential meteorological covariates, including daily relative humidity, daily precipitation, and daily temperature as lag‐specific continuous variables corresponding to the same lag structure as the exposure of interest.

This study also conducted comprehensive stratified analyses to examine whether the association between wildfire smoke PM_2.5_ exposure and asthma exacerbations in adults varied across demographic groups, geographic regions, and time periods. Stratifications were performed by age group (18–34, 35–54, 55–64, and ≥65 years), sex (female, male, and unknown), race/ethnicity (non‐Hispanic White, non‐Hispanic Black, Hispanic, non‐Hispanic others, and unknown), residential region (midwestern, northeastern, southeastern, southwestern, and western USA; see Figure S1, Table S10 in Supporting Information S1), and month (April–August). We also conducted two sensitivity analyses to assess the influence of recurrent exacerbations. First, we refitted the conditional logistic regression models using robust standard errors clustered by participant to account for within‐person correlation across repeated exacerbation events. Second, we restricted the case‐crossover data set to each participant's first recorded exacerbation during the study period and its corresponding matched control periods (*n* = 6,154 participants).

## 3 Results

Between 2006 and 2019, during the warm months (April–August), a total of 18,935 asthma exacerbations were recorded among 6,154 adults who experienced at least one exacerbation during the study period (Table 1). The median number of exacerbations per adult was 1 (interquartile range [IQR]: 2), with a maximum of 45 exacerbations per individual. Notably, 56.9% of adults in the study population experienced only one exacerbation (Table 1). Among the 18,935 exacerbations, the majority occurred in non‐Hispanic White (42.0%) and non‐Hispanic Black (32.2%) adults; females accounted for 74.8% of the total exacerbations, and 42.8% occurred in individuals aged 35–54 years. Geographically, 31.1% and 36.1% of exacerbations occurred in the midwestern and northeastern USA, respectively. Seasonal trends indicated that these asthma exacerbations were most frequent in April (24.1%) and least frequent in July (16.4%) (Table 1). Detailed descriptive statistics of wildfire PM_2.5_ and meteorological variables are provided in Table S9 of Supporting Information S1, and maps showing the percentage of days with wildfire smoke PM_2.5_ > 0 μg/m^3^ and the mean concentration among exposed days (PM_2.5_ > 0 μg/m^3^), by 3‐digit ZIP code region for 2006–2019, are provided in Figure S3 of Supporting Information S1.

**Table 1:** Characteristics of Asthma Exacerbation in Adults in Electronic Health Records From the National Institutes of Health (NIH) *All of Us* Research Program Across the Contiguous USA, April–August 2006–2019

Characteristics	Count (%)
Total asthma exacerbations	18,935
Total adults	6,154
Exacerbations per adult	
Median (IQR)	1 (2)
Maximum	45
N adult (% of 6154 total adults)	
One exacerbation	3,502 (56.9)
Two exacerbations	835 (13.6)
Three exacerbations	479 (7.8)
Four exacerbations	314 (5.1)
Five exacerbations	217 (3.5)
More than five exacerbations	807 (13.1)
Race/ethnicity (n [% of total exacerbations])	
Non‐Hispanic White	7,954 (42.0)
Non‐Hispanic Black	6,094 (32.2)
Hispanic	3,352 (17.7)
Non‐Hispanic Others	610 (3.2)
Unknown	925 (4.9)
Age (n [% of total exacerbations])	
18‐34	3,137 (16.6)
35‐54	8,107 (42.8)
55‐64	4,495 (23.7)
≥65	3,196 (16.9)
Sex (n [% of total exacerbations])	
Male	4,359 (23.0)
Female	14,163 (74.8)
Unknown	413 (2.2)
Residential region (n [% of total exacerbations])	
Midwest	5,883 (31.1)
Northeast	6,830 (36.1)
Southeast	2,458 (13.0)
Southwest	2,730 (14.4)
West	1,034 (5.5)
Visit month (n [% of total exacerbations])	
April	4,569 (24.1)
May	4,310 (22.8)
June	3,613 (19.1)
July	3,112 (16.4)
August	3,331 (17.6)

*Note.* SD: Standard deviation. IQR: Interquartile range.

Wildfire smoke PM_2.5_ exposure was associated with significantly higher odds of adult asthma exacerbation across multiple lag periods throughout the contiguous USA during the warm months (April–August) from 2006 to 2019 (Table 2). We estimated adjusted odds ratios (ORs) and corresponding 95% confidence intervals (CIs) for asthma exacerbation odds associated with wildfire smoke PM_2.5_ exposure on the day of the event (0‐day lag) and for each of the seven preceding days (1‐day to 7‐day lag). At lag 0, 18.6% of case observations and 12.3% of control observations were exposed. The strongest association was observed at a 2‐day lag (adjusted OR: 1.38; 95% CI: 1.32–1.44), indicating that exposure to wildfire smoke PM_2.5_ 2 days prior was associated with a 38% increase in the odds of asthma exacerbation compared with the unexposed reference group. Elevated and statistically significant odds were observed across all lag periods, with adjusted ORs ranging from 1.27 (0‐day lag) to 1.38 (2‐day lag).

**Table 2:** Adjusted Odds Ratio (Adjusted OR) for the Association Between Wildfire Smoke PM_2.5_ Exposure and Asthma Exacerbation in Adults, Across 0‐ to 7‐Day Lags, in the Contiguous USA During April–August 2006–2019

No. of days before exacerbation	No. of exposed cases	No. of unexposed case	No. of exposed controls	No. of unexposed controls	Adjusted OR^a^ (95% CIs)
0	3,521	15,414	9,333	66,407	1.27 (1.22, 1.33) ^b^
1	3,621	15,314	9,239	66,501	1.35 (1.29, 1.41) ^b^
2	3,615	15,320	9,052	66,688	1.38 (1.32, 1.44) ^b^
3	3,511	15,424	9,119	66,621	1.31 (1.25, 1.36) ^b^
4	3,514	15,421	9,026	66,714	1.30 (1.24, 1.36) ^b^
5	3,449	15,486	8,732	67,008	1.34 (1.28, 1.40) ^b^
6	3,416	15,519	8,829	66,911	1.29 (1.25, 1.33) ^b^
7	3,310	15,625	8,592	67,148	1.30 (1.25, 1.36) ^b^

^a^ Adjusted for daily mean temperature, daily mean surface precipitation amount, and daily mean relative humidity.

^b^ Statistically significant after Bonferroni correction for multiple comparisons at level 0.05.

Figure 1 presents the results of the stratified analyses examining the association between wildfire smoke PM_2.5_ exposure (0‐day to 7‐day lag) and asthma exacerbations among adults of different age groups (18–34, 35–54, 55–64, and ≥65 years) across the contiguous USA during the warm months (April–August) from 2006 to 2019. The strongest association was observed in adults aged ≥65 years at 2‐day lag, with an adjusted OR of 1.36 (95% CI: 1.21–1.53), indicating that exposure to wildfire smoke PM_2.5_ two days prior was associated with 36% higher odds of an asthma exacerbation in this age group. Notably, the adjusted ORs for adults aged ≥65 years remained the highest among all age groups from 0‐ to 2‐day lags. The 55–64 age group exhibited consistently significant positive associations across all lags from 0 to 7 days. The associations generally increased over time, although slight decreases were observed at the 4‐ and 6‐day lags. Among younger adults aged 18–34 years, the strongest association was observed at the 4‐day lag (adjusted OR: 1.32; 95% CI: 1.17–1.49). Overall, the younger age groups exhibited lower adjusted ORs compared to older adults during the shorter lag periods (0‐ to 2‐ day lags).

**Figure 1:**
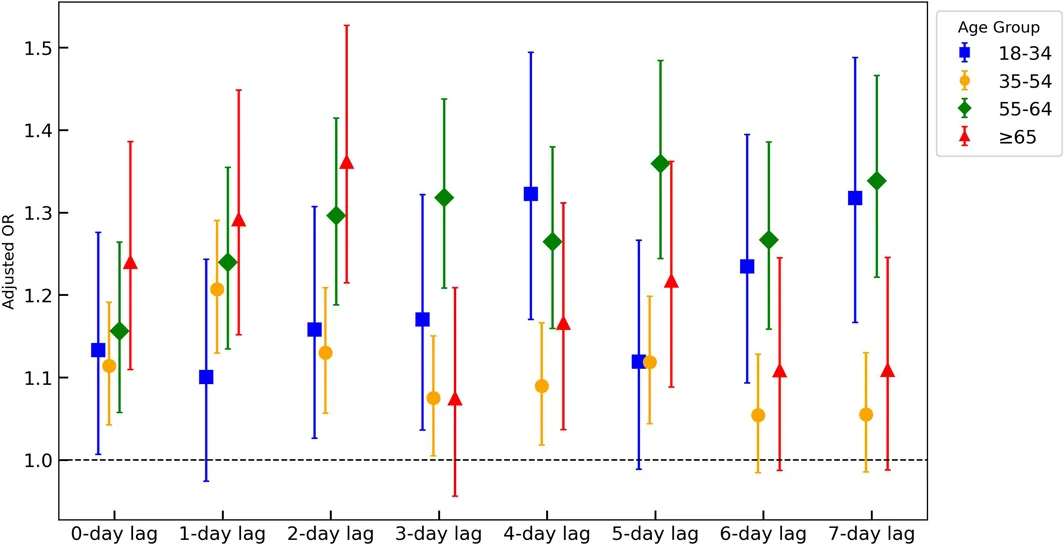
Adjusted odds ratio (adjusted OR) for the association between wildfire smoke PM_2.5_ exposure and asthma exacerbation in adults, across 0‐ to 7‐day lags, in the contiguous USA during April–August 2006–2019; stratified by age group.

Figure 2 presents sex‐stratified associations between wildfire smoke PM_2.5_ exposure and asthma exacerbations. Among adult females, the highest adjusted OR was observed with exposure 2 days prior to exacerbations (2‐day lag), with an adjusted OR of 1.25 (95% CI: 1.19–1.33), indicating a 25% increase in the odds of asthma exacerbation associated with wildfire smoke PM_2.5_. Among adult males, the highest OR was found with exposure one day prior to exacerbations (1‐day lag), with an adjusted OR of 1.30 (95% CI: 1.20–1.40), suggesting 30% increased odds of asthma exacerbation associated with wildfire smoke PM_2.5_ exposure at this lag. Overall, the associations between wildfire smoke PM_2.5_ exposure and asthma exacerbations were similar for adult females and males, with both groups experiencing a significant increase in asthma exacerbation odds following exposure.

**Figure 2:**
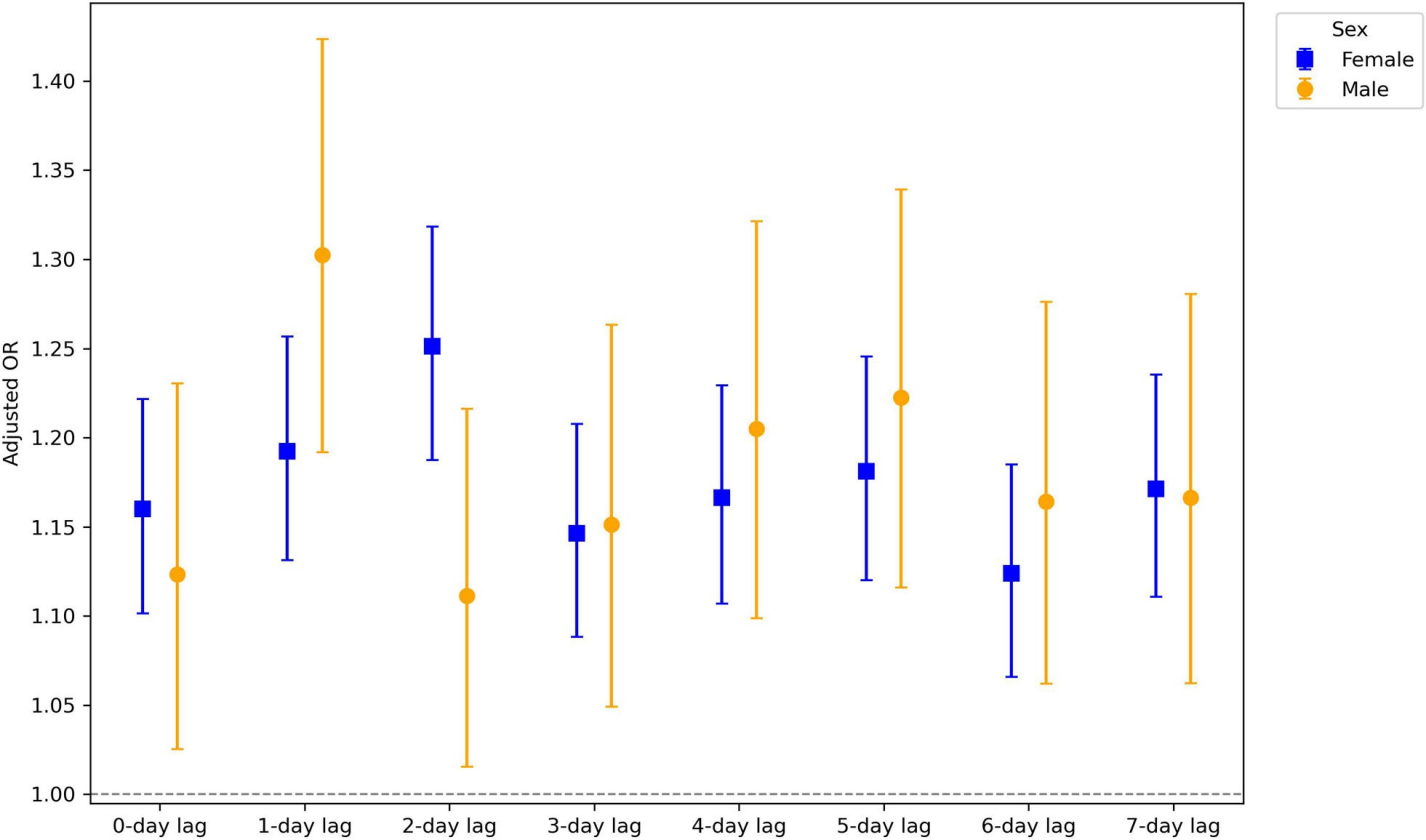
Adjusted odds ratio (adjusted OR) for the association between wildfire smoke PM_2.5_ exposure and asthma exacerbation in adults, across 0‐ to 7‐day lags, in the contiguous USA during April–August 2006–2019; stratified by sex.

Figure 3 illustrates the associations between wildfire smoke PM_2.5_ exposure and asthma exacerbations, stratified by race/ethnicity. The highest adjusted OR of 1.39 (95% CI: 1.29–1.49) was observed at 1‐day lag among non‐Hispanic White individuals, indicating a 39% increase in the odds of asthma exacerbation following exposure one day prior. Across all lag periods, non‐Hispanic White individuals consistently exhibited significant positive associations, with the highest adjusted ORs among all racial/ethnic groups except at the 6‐day lag. For non‐Hispanic Black individuals, the strongest association occurred at the 4‐day lag (adjusted OR:1.19; 95% CI: 1.10–1.28). Positive associations were observed at all lag periods, though those at the 0‐ and 1‐day lags were not statistically significant. Among Hispanic adults, the strongest association was at the 3‐day lag (adjusted OR: 1.16; 95% CI: 1.03–1.30), while other lag periods showed no significant associations. Additionally, non‐Hispanic individuals categorized as “other” exhibited no significant associations at any lag.

**Figure 3:**
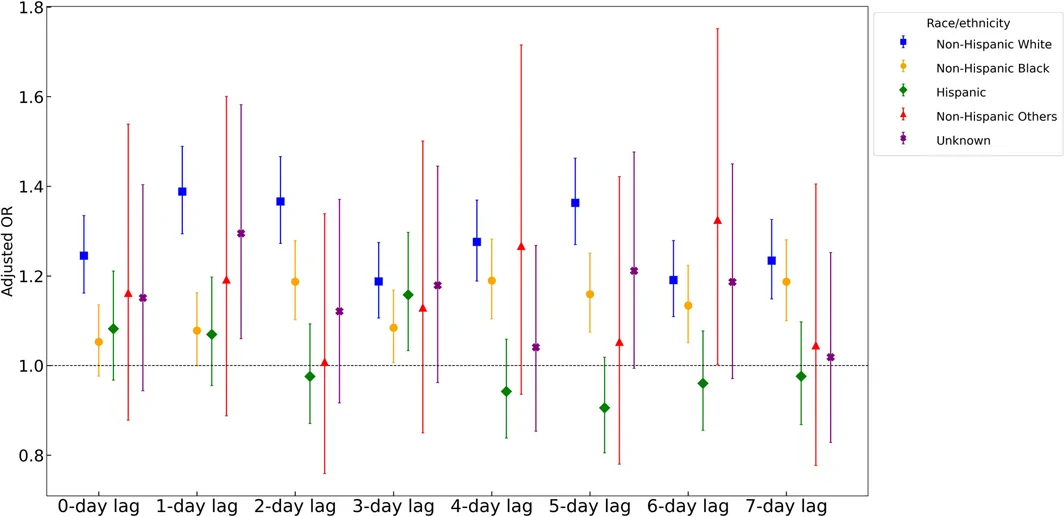
Adjusted odds ratio (adjusted OR) for the association between wildfire smoke PM_2.5_ exposure and asthma exacerbation in adults, across 0‐ to 7‐day lags, in the contiguous USA during April–August 2006–2019; stratified by race/ethnicity.

Figure 4 compares the associations between wildfire smoke PM_2.5_ exposure and asthma exacerbations among five regions of the USA. The highest adjusted OR in the midwestern USA was observed at the 5‐day lag (adjusted OR: 1.60; 95% CI: 1.48–1.72), indicating a 60% increase in the odds of asthma exacerbation following exposure to wildfire smoke PM_2.5_ 5 days prior. Notably, all lag periods in the midwestern USA exhibited significant positive associations. In contrast, the southwestern USA exhibited a significant association only at the 1‐day lag (adjusted OR: 1.16; 95% CI: 1.03–1.30), while in the western USA a significant association was identified at the 7‐day lag (adjusted OR: 1.34; 95% CI: 1.06–1.71). Overall, associations between wildfire smoke PM_2.5_ exposure and asthma exacerbations were stronger and more consistent in the midwestern USA than in the other regions.

**Figure 4:**
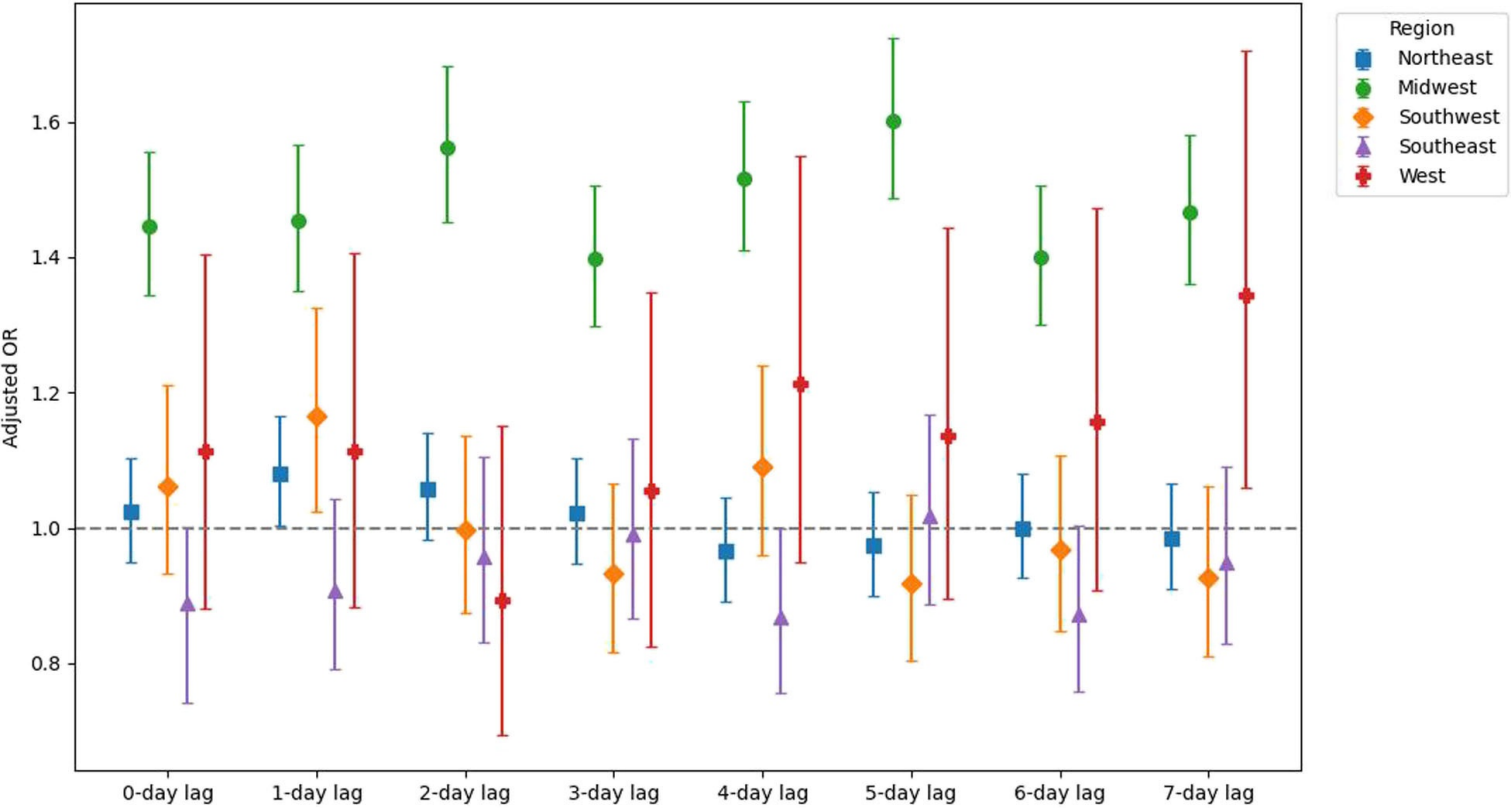
Adjusted odds ratio (adjusted OR) for the association between wildfire smoke PM_2.5_ exposure and asthma exacerbation in adults, across 0‐ to 7‐day lags, in the contiguous USA during April–August 2006–2019; stratified by region.

Figure 5 illustrates the associations between wildfire smoke PM_2.5_ exposure and asthma exacerbations stratified by month. The strongest association was observed in May at a 5‐day lag (adjusted OR: 1.63; 95% CI: 1.46–1.84), indicating that exposure 5 days prior was associated with a 63% increase in the odds of asthma exacerbation compared with the unexposed reference group. Notably, May exhibited consistently significant positive associations across all lags. April showed significant positive associations at the 2‐day lag (adjusted OR: 1.22; 95% CI: 1.06–1.41) and 7‐day lag (adjusted OR: 1.27; 95% CI: 1.10–1.47). June demonstrated moderate associations, peaking at the 1‐day lag (adjusted OR: 1.26; 95% CI: 1.13–1.39). July consistently displayed significant positive associations, with a peak at the 2‐day lag (adjusted OR: 1.32; 95% CI: 1.21–1.44). Similarly, August exhibited predominantly significant positive associations across all lags, except at the 3‐day lag, with the highest adjusted OR at the 1‐day lag (adjusted OR: 1.29; 95% CI: 1.18–1.40). Across all months, the 2‐day lag consistently showed significant positive associations, suggesting a potential delayed effect of wildfire smoke PM_2.5_ exposure on asthma exacerbations during April to August.

**Figure 5:**
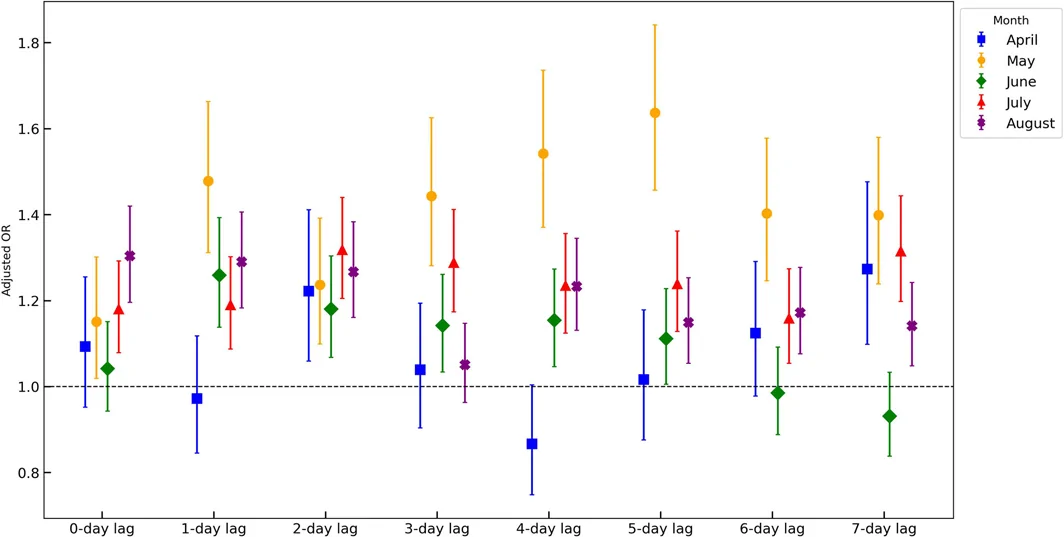
Adjusted odds ratio (adjusted OR) for the association between wildfire smoke PM_2.5_ exposure and asthma exacerbation in adults, across 0‐ to 7‐day lags, in the contiguous USA during April–August 2006–2019; stratified by month.

## 4 Discussion

Our findings suggest that exposure to wildfire smoke PM_2.5_ within the preceding week is significantly associated with increased odds of asthma exacerbation in adults. Subgroup analyses suggest that older adults, non‐Hispanic White individuals, residents of the midwestern USA, and those exposed during the month of May experience particularly elevated odds of asthma exacerbation in response to wildfire smoke PM_2.5_ exposure.

These findings align with the existing literature, reinforcing the significant impact of PM_2.5_ exposure on asthma exacerbations and respiratory health across diverse populations and geographic regions. Biologically, short‐term PM_2.5_ exposure has been linked to genome‐wide DNA methylation changes, which may contribute to airway inflammation, immune response dysregulation, and airway remodeling, mechanisms that can potentially worsen asthma symptoms (Duan et al., 2024). Acute pollution events, such as wildfires, have been shown to trigger significant increases in asthma exacerbations. For example, asthma‐related ED visits were 17% higher than expected during the 19 days of wildfire smoke in the United States, between April 30 and 4 August 2023, when smoke from Canadian wildfires affected air quality (McArdle et al., 2023). A previous study also found that three‐month exposure to smoke‐related PM_2.5_ was associated with increased hospitalization risks for most cardiorespiratory diseases across the United States between 2006 and 2016 (Wei et al., 2025). Similarly, during a wildfire that began over the weekend of June 20–21, 2008, in northern California, USA, exposure to PM_2.5_ two days prior (OR: 1.112; 95% CI: 1.087–1.138) was significantly associated with increased respiratory ED visits and hospitalizations (Reid et al., 2019). Looking ahead, projections suggest that rising wildfire smoke PM_2.5_ concentrations will significantly increase asthma‐related emergency visits and hospitalizations, particularly in the western USA, leading to considerable public health and economic burdens (Stowell et al., 2022). These findings are consistent with the growing body of literature linking short‐term PM_2.5_ exposure to adverse respiratory outcomes, further supporting the results of the present study.

Table 2 shows that the ORs varied across different lag periods, reflecting the complex and delayed physiological responses to wildfire smoke PM_2.5_ exposure. The strongest association was observed at the 2‐day lag, suggesting that exposure 2 days prior carries the highest odds for asthma exacerbation. This finding aligns with previous studies reporting a similar temporal pattern of association (Reid et al., 2019; Zheng et al., 2015). This delay is also biologically plausible, as airway inflammation and immune responses typically peak within 24–72 hr following particulate matter exposure, leading to a delayed onset of clinical asthma exacerbations (Wegesser & Last, 2008; Wu et al., 2018; Xing et al., 2016).

Based on the results from the stratified analysis, although wildfires predominantly occur in the western USA, where PM_2.5_ emissions from fire smoke are more frequent and intense, our findings show that midwestern USA has the most consistent positive associations across lag days, while other regions demonstrated weaker associations. Although wildfire PM_2.5_ emissions are generally more frequent and intense in the western USA, higher odds ratios in the Midwest do not necessarily indicate higher absolute wildfire smoke concentrations. Instead, these regional differences may reflect several factors, including long‐range transport of wildfire smoke, regional topographic conditions that influence smoke dispersion and persistence, baseline asthma burden, health care access and utilization, exposure timing, and population susceptibility. The stratified analysis by race/ethnicity showed lower and less consistent associations among Hispanic adults compared with some other groups. This pattern should not be interpreted as evidence of lower biological susceptibility. Instead, it may reflect differences in sample size, geographic distribution, health care access, EHR capture, exposure patterns, asthma diagnosis or treatment, and residual confounding by social and environmental factors. Additional studies with finer geographic information, more detailed socioeconomic and clinical variables, and larger subgroup‐specific samples are needed to better understand racial and ethnic differences in wildfire smoke‐related asthma exacerbation risk. Stratified demographic analysis further revealed that older adults were more susceptible to wildfire smoke‐related asthma exacerbations. This increased vulnerability may reflect age‐related declines in lung function, the presence of pre‐existing respiratory conditions, and reduced physiological resilience to airborne pollutants, which may contribute to stronger or more rapid clinical responses following wildfire smoke exposure (US EPA, 2021b). In addition to having higher odds ratios, older adults also tended to experience symptoms more rapidly than individuals younger than 35, suggesting a shorter lag period between exposure and clinical exacerbation. Among the months studied, May exhibited the highest adjusted ORs for asthma exacerbations following wildfire smoke PM_2.5_ exposure. This may reflect several contributing factors: May marks the seasonal transition from spring to summer, a period of increasing wildfire activity (US EPA, 2025), and coincides with increased outdoor activity due to warmer weather, which may elevate the risk of inhaling wildfire smoke pollutants. While seasonal allergens such as pollen may also contribute to asthma exacerbations during this period, the case‐crossover design inherently controls for seasonality, and our models further adjusted for meteorological and environmental covariates to help isolate the effect of wildfire smoke. Together, these factors support the conclusion that the associations observed in May are primarily attributable to wildfire smoke PM_2.5_ exposure. Lastly, in the sex‐stratified analysis, we observed broadly similar positive associations among females and males, suggesting limited evidence of substantial sex‐based effect modification in this study population. This finding is generally consistent with the broader wildfire‐smoke literature showing that respiratory risks are elevated in both sexes, although some studies have reported stronger associations among females. For example, a meta‐analysis of wildfire smoke and respiratory health in North America reported modestly greater wildfire‐smoke effects among females than males for asthma‐related outcomes (Kondo et al., 2019). Recent analyses of wildfire smoke‐related emergency department visits in the western United States also reported higher asthma risks among females than males (Chen et al., 2023). In contrast, our estimates for females and males were similar in magnitude across most lag days, which may reflect differences in study population, geographic coverage, age distribution, and health care utilization patterns. Therefore, we interpret the lack of substantial sex‐based differences cautiously.

To assess the stability and consistency of our findings, we conducted a series of sensitivity analyses. First, we examined exposure status over cumulative lag periods of 0–1, 0–2, 0–3, 0–4, 0–5, 0–6, and 0–7 days preceding each case date and its matched controls. Table S1 in Supporting Information S1 summarizes the associations between wildfire smoke PM_2.5_ exposure during these lag periods and asthma exacerbations. The strongest association was observed at the (0–7)‐day cumulative lag (adjusted OR: 1.41; 95% CI: 1.36–1.46), suggesting that exposure to wildfire smoke PM_2.5_ on any day within the preceding week is associated with 41% higher odds of asthma exacerbation compared with adults without such exposure. The increased odds remained statistically significant across all lag periods, ranging from 1.30 for (0–1)‐day lag to 1.41 for (0–7)‐day lag. Second, we conducted sensitivity analyses stratified by exacerbation frequency, restricting the data set separately to individuals with only a single recorded exacerbation (*n* = 3,502) and to those with multiple recorded exacerbations (*n* = 2,652) in the *All of Us* database, along with their corresponding matched control periods. As shown in Table S2 and Table S3 in Supporting Information S1, the associations remained largely consistent with the main results in Table 2 across both strata. These findings suggest that the observed relationship between wildfire PM_2.5_ exposure and asthma exacerbation is robust to differences in exacerbation frequency. Third, the analysis using participant‐clustered standard errors yielded associations consistent in direction and magnitude with the primary analysis (Table S4 in Supporting Information S1). In the first‐event‐only analysis, associations remained positive across lags 0–7 days, but the odds ratios were attenuated compared with the primary analysis (Table S5 in Supporting Information S1). These findings support the consistency of the positive associations while indicating that their magnitude is sensitive to the inclusion of recurrent exacerbations. Fourth, we performed an additional sensitivity analysis modeling wildfire smoke PM_2.5_ exposure as a continuous variable, based on the average concentration within each three‐digit ZIP code during the corresponding exposure window. We estimated adjusted OR per 1 μg/m^3^ increase in wildfire smoke PM_2.5_ across all lag periods. The results (Table S6 in Supporting Information S1) were consistent with the main findings. Specifically, higher wildfire smoke PM_2.5_ concentrations were associated with increased odds of asthma exacerbation across lag days 0–7. The binary metric was useful for distinguishing days with any detectable wildfire smoke PM_2.5_ from days without wildfire smoke, whereas the continuous metric captured variation in exposure intensity and allowed comparison with studies reporting effects per concentration increment. Together, these findings suggest that the observed association was not dependent on the binary exposure definition. Fifth, we additionally adjusted the models for co‐pollutants, including daily NO_2_ and O_3_ concentrations (US EPA, 2021a), to assess potential confounding by other ambient air pollutants. The inclusion of these co‐pollutants did not materially change the estimated associations between wildfire smoke PM_2.5_ exposure and asthma exacerbations, supporting the robustness of the main findings (Table S7 in Supporting Information S1). Finally, to further assess potential time‐trend bias, we conducted a sensitivity analysis stratified by calendar year from 2006 to 2019. The associations between wildfire smoke PM_2.5_ exposure and asthma exacerbations were generally consistent across 2016 to 2018 (Figure S2 in Supporting Information S1), indicating that the main findings were robust to interannual variation in wildfire activity and exposure levels. Overall, the sensitivity analyses support the consistency of the positive association between wildfire smoke PM_2.5_ exposure and asthma exacerbations, although the attenuation in the first‐event‐only analysis indicates that the estimated magnitude varies with the inclusion of recurrent events.

This study also has several limitations. First, only three‐digit ZIP code information was available for each participant's residential location, as provided by the *All of Us* database. This coarse spatial resolution may have introduced exposure misclassification because wildfire smoke PM_2.5_ exposure was assigned using ZIP‐level averages of 10‐km grid cells. Because three‐digit ZIP areas can be large, especially in rural Western regions, this approach may not capture within‐ZIP variation in wildfire smoke PM_2.5_, particularly where smoke gradients are steep due to fire proximity, topography, or meteorological conditions. Such exposure misclassification may bias the estimated associations, but the direction and magnitude of bias are uncertain because exposure errors may be spatially heterogeneous and differential. This unweighted ZIP‐level average may not fully capture within‐ZIP variation in wildfire smoke PM_2.5_, especially in large rural ZIP areas where grid cells may include sparsely populated or uninhabited land such as forests, mountains, or remote terrain. As a result, ZIP‐level average exposure may differ from the exposure actually experienced by participants. Population‐weighted exposure assignment would be an important improvement for future studies when compatible gridded population data and finer residential information are available. While this limitation stems from privacy regulations aimed at protecting participant confidentiality, it reflects the inherent trade‐off between data accessibility and individual confidentiality. Although the three‐digit ZIP codes may capture broader geographic patterns of human mobility, using finer‐scale residential data could improve the precision of exposure assessment in future studies. In addition, the *All of Us* Research Program is not a probability‐based sample of the U.S. adult population. Because participants are recruited through health care provider organizations and community‐based enrollment, and because this analysis required available EHR records and residential three‐digit ZIP code information, the study population may differ from the broader U.S. adult population in demographics, health care access and utilization, and geographic distribution. These factors may limit generalizability. Therefore, our findings should be interpreted as most directly applicable to adults represented in *All of Us* with available EHR and geographic information, while still providing broader geographic coverage than many prior single‐state or single‐event wildfire smoke studies.

Second, our use of exclusively preceding control days may have introduced temporal bias. We selected four same‐weekday control days within the 4 weeks before each exacerbation to avoid referent periods potentially affected by treatment or behavioral changes following the exacerbation‐related clinical visit. However, this approach does not eliminate potential bias if wildfire smoke exposure or underlying exacerbation risk changes systematically over the referent period. The direction and magnitude of this potential bias are uncertain, and our calendar‐year‐stratified analyses did not directly assess short‐term temporal trends. Although we adjusted for temperature, relative humidity, and precipitation, residual confounding by seasonal factors, including pollen concentrations and circulating respiratory viruses, cannot be excluded. Future studies should evaluate sensitivity to referent selection using a time‐stratified case‐crossover design with same‐weekday control dates within the same calendar month and year and incorporate information on other short‐term asthma triggers.

Third, the observed lag effects may be influenced by variations in healthcare access or the operational schedules of medical facilities (e.g., limited hours or weekend closures), which could affect the timing of asthma exacerbation diagnoses or reporting. Incorporating data on healthcare utilization and accessibility in future analyses could improve the accuracy of estimated lag effects and provide a more nuanced understanding of temporal associations. Additionally, this study lacks information on baseline use of asthma controller medications. Because controller medication use is an important marker of underlying disease severity and may influence susceptibility to exacerbations following environmental exposures, the absence of this information may introduce uncertainty in the interpretation of the results.

Finally, this study focused on April‐August as a consistent national warm‐season window to align with the primary wildfire period and preserve exposure contrast within the case‐crossover design. However, this window may not capture regional wildfire seasons equally. In particular, severe wildfire smoke events in the western USA often extend into September, October, or November. Excluding these later months may underrepresent wildfire smoke exposure in the West and may affect interpretation of regional comparisons. Therefore, estimates for the western USA should be interpreted as warm‐season associations rather than full wildfire‐season associations. Future studies should evaluate region‐specific wildfire seasons, including extended fall wildfire periods in the West.

## 5 Conclusion

This study demonstrates that wildfire smoke PM_2.5_ exposure increases the odds of asthma exacerbations among adults in the U.S., with spatial, temporal, and demographic variability in susceptibility. These findings highlight the importance of exposure time windows and population vulnerability for public health. Future research should (a) incorporate finer‐scale exposure assessment and additional seasonal triggers such as pollen and respiratory infections, (b) evaluate mechanisms underlying regional differences in susceptibility, and (c) examine interventions during high‐risk periods, particularly in late spring and summer. These efforts will help refine public health early‐warning systems and guide region‐specific mitigation strategies to reduce asthma morbidity during wildfire seasons.

## Conflict of Interest

The authors declare no conflicts of interest relevant to this study.

## Supporting information

Supporting Information S1

## Data Availability

The health data used in this study was obtained from the NIH *All of Us* Research Program and are not publicly available due to access restrictions. Qualified researchers may request access through the *All of Us* Researcher Workbench, subject to program requirements and approval. The wildfire smoke PM_2.5_ data used for the exposure in this study are available (Childs et al., 2022). Daily meteorological data across the USA during 2006–2019 was obtained from the NOAA's National Center for Environmental Prediction (NCEP) North American Regional Reanalysis (NARR) data set (https://psl.noaa.gov/data/gridded/data.narr.html).
