# Supplementary material for: Asthma Exacerbations Following Wildfire Smoke PM2.5 Exposure Among U.S. Adults
Source: Geohealth. 2026 Nov 7;10(11):e2026GH001972. doi: 10.1029/2026GH001972 (PMC13744005; doi:10.1029/2026GH001972)
Supplement: Supplementary file 1 [file GH2-10-e2026GH001972-s001.docx]

***GeoHealth***

**Supplementary Material for**

**Asthma Exacerbations Following Wildfire Smoke PM_2.5_ Exposure Among U.S. Adults**

**Yanhong Huang**

Department of Veterinary Physiology and Pharmacology, Texas A&M University, College Station, Texas, 77843, USA

Department of Biobehavioral Health, The Pennsylvania State University, University Park, Pennsylvania, 16802, USA

Department of Geography & Environmental Studies, University of New Mexico, Albuquerque, New Mexico, 87131, USA

UNM Center for the Advancement of Spatial Informatics Research and Education (ASPIRE), University of New Mexico, Albuquerque, New Mexico, 87131, USA

([yanhonghuang@tamu.edu](mailto:yanhonghuang@tamu.edu))

**Li Luo**

Department of Internal Medicine, University of New Mexico, Albuquerque, New Mexico, 87131, USA

University of New Mexico Comprehensive Cancer Center, Albuquerque, New Mexico, 87131, USA

([LLuo@salud.unm.edu](mailto:LLuo@salud.unm.edu))

**Shuguang Leng**

Department of Internal Medicine, University of New Mexico, Albuquerque, New Mexico, 87131, USA

University of New Mexico Comprehensive Cancer Center, Albuquerque, New Mexico, 87131, USA

Lung Cancer Program, Lovelace Biomedical Research Institute, Albuquerque, New Mexico, 87131, USA

([SLeng@salud.unm.edu](mailto:SLeng@salud.unm.edu))

**Su Zhang**

Department of Geography & Environmental Studies, University of New Mexico, Albuquerque, New Mexico, 87131, USA

UNM Center for the Advancement of Spatial Informatics Research and Education (ASPIRE), University of New Mexico, Albuquerque, New Mexico, 87131, USA

([suzhang@unm.edu](mailto:suzhang@unm.edu))

**Roberta P Lavin**

Center for Health Equity and Preparedness, College of Nursing, University of New Mexico, Albuquerque, New Mexico, 87131, USA ([RLavin@salud.unm.edu](mailto:RLavin@salud.unm.edu))

**Michaela Buenemann**

Department of Geography, New Mexico State University, Las Cruces, New Mexico, 88003, USA ([elabuen@nmsu.edu](mailto:elabuen@nmsu.edu))

**Yan Lin**

Department of Geography, The Pennsylvania State University, University Park, PA 16802, USA

Social Science Research Institute (SSRI), The Pennsylvania State University, University Park, PA 16802, USA

([yanlin@psu.edu](mailto:yanlin@psu.edu))

**Matthew Campen**

Department of Pharmaceutical Sciences, College of Pharmacy, University of New Mexico, Albuquerque, NM, 87131, USA

([Mcampen@salud.unm.edu](mailto:Mcampen@salud.unm.edu))

**Xi Gong***

Department of Biobehavioral Health, Institute for Computational and Data Sciences (ICDS), The Pennsylvania State University, University Park, Pennsylvania, 16802, USA

([xigong@psu.edu](mailto:xigong@psu.edu))

* Corresponding author

**Table of contents**

Table S1. Adjusted odds ratio (adjusted OR) for the association between wildfire smoke PM2.5 exposure and asthma exacerbation in adults, across (0-1)-day to (0-7)-day lags, in the contiguous USA during April–August, 2006–2019.

Table S2. Adjusted odds ratio (adjusted OR) for the association between wildfire smoke PM2.5 exposure and asthma exacerbation in adults with only one exacerbation, across 0- to 7-day lags, in the contiguous USA during April–August, 2006–2019.

Table S3. Adjusted odds ratio (adjusted OR) for the association between wildfire smoke PM2.5 exposure and asthma exacerbation in adults with multiple exacerbations, across 0- to 7-day lags, in the contiguous USA during April–August, 2006–2019.

Table S4. Adjusted odds ratio (adjusted OR) for the association between wildfire smoke PM2.5 exposure and asthma exacerbation in adults, across 0- to 7-day lags, in the contiguous USA during April–August, 2006–2019, accounting for cluster standard errors.

Table S5. Adjusted odds ratio (adjusted OR) for the association between wildfire smoke PM2.5 exposure and asthma exacerbation in adults with the first exacerbation, across 0- to 7-day lags, in the contiguous USA during April–August, 2006–2019.

Table S6. Adjusted odds ratio (adjusted OR) for the association between wildfire smoke PM2.5 exposure (per 1ug/m3 increase), across 0- to 7-day lags, in the contiguous USA during April–August, 2006–2019.

Table S7. Adjusted odds ratio (adjusted OR) for the association between wildfire smoke PM2.5 exposure and asthma exacerbation in adults, across 0- to 7-day lags, adjusting for co-pollutants, in the contiguous USA during April–August, 2006–2019.

Table S8. Condition name and code for asthma exacerbation.

Table S9. Descriptive statistics of meteorological variables and ambient air pollutants at lag days preceding asthma exacerbation events.

Table S10. Distribution of 3-Digit ZIP Code Land Area by U.S. Region.

Fig. S1. Five USA state regions for stratified analysis

Fig. S2. Adjusted odds ratio (adjusted OR) for the association between wildfire smoke PM_2.5_ exposure and asthma exacerbation in adults, across 0- to 7-day lags, in the contiguous USA during April–August, 2006–2019; stratified by year

Fig. S3. Spatial distribution of wildfire smoke PM2.5 exposure by 3-digit ZIP code (ZIP3) region in the contiguous USA during April–August, 2006–2019. (a) Percentage of days with wildfire PM2.5 > 0 µg/m³. (b) Mean wildfire PM2.5 concentration (µg/m³) among days with PM2.5 > 0.

**Table S1.** Adjusted odds ratio (adjusted OR) for the association between wildfire smoke PM_2.5_ exposure and asthma exacerbation in adults, across (0-1)-day to (0-7)-day lags, in the contiguous USA during April–August, 2006–2019.

| Range of days before exacerbation | No. of exposed case | No. of unexposed case | No. of exposed control | No. of unexposed control | Adjusted OR^a^ (95% CIs) |
| --- | --- | --- | --- | --- | --- |
| 0-1 | 5,124 | 13,811 | 16,304 | 59,436 | 1.30 (1.25, 1.35) * |
| 0-2 | 6,335 | 12,600 | 20,344 | 55,396 | 1.33 (1.28, 1.38) * |
| 0-3 | 7,259 | 11,676 | 23,723 | 52,017 | 1.34 (1.29, 1.39) * |
| 0-4 | 8,057 | 10,878 | 26,758 | 48,982 | 1.33 (1.29, 1.38) * |
| 0-5 | 8,739 | 10,196 | 29,112 | 46,628 | 1.35 (1.30, 1.40) * |
| 0-6 | 9,376 | 9,559 | 31,001 | 44,739 | 1.37 (1.32, 1.42) * |
| 0-7 | 9,929 | 9,006 | 33,096 | 42,644 | 1.41 (1.36, 1.46) * |

^a^Adjusted for daily mean temperature, daily mean surface precipitation amount, and daily mean relative humidity.

^* Statistically significant after Bonferroni correction for multiple comparisons at level 0.05.^

**Table S2.** Adjusted odds ratio (adjusted OR) for the association between wildfire smoke PM_2.5_ exposure and asthma exacerbation in adults with only one exacerbation, across 0- to 7-day lags, in the contiguous USA during April–August, 2006–2019.

|  | No. of days before exacerbation | No. of exposed case | | No. of unexposed case | No. of exposed control | No. of unexposed control | Adjusted OR^a^ (95% CIs) |
| --- | --- | --- | --- | --- | --- | --- | --- |
| 0 | | | 436 | 3,066 | 1,341 | 12,667 | 1.22 (1.08, 1.37) * |
| 1 | | | 453 | 3,049 | 1,338 | 12,670 | 1.34 (1.18, 1.51) * |
| 2 | | | 457 | 3,045 | 1,213 | 12,795 | 1.46 (1.28, 1.65) * |
| 3 | | | 439 | 3,063 | 1,258 | 12,750 | 1.34 (1.18, 1.52) * |
| 4 | | | 454 | 3,048 | 1,306 | 12,702 | 1.41 (1.25, 1.58) * |
| 5 | | | 450 | 3,052 | 1,202 | 12,806 | 1.43 (1.26, 1.61) * |
| 6 | | | 412 | 3,090 | 1,207 | 12,801 | 1.25 (1.10, 1.41) * |
| 7 | | | 435 | 3,067 | 1,235 | 12,773 | 1.39 (1.23, 1.56) * |

^a^Adjusted for daily mean temperature, daily mean surface precipitation amount, and daily mean relative humidity.

^* Statistically significant after Bonferroni correction for multiple comparisons at level 0.05.^

**Table S3.** Adjusted odds ratio (adjusted OR) for the association between wildfire smoke PM_2.5_ exposure and asthma exacerbation in adults with multiple exacerbations, across 0- to 7-day lags, in the contiguous USA during April–August, 2006–2019.

|  | No. of days before exacerbation | No. of exposed case | | No. of unexposed case | No. of exposed control | No. of unexposed control | Adjusted OR^a^ (95% CIs) |
| --- | --- | --- | --- | --- | --- | --- | --- |
| 0 | | | 3,085 | 12,348 | 7,992 | 53,740 | 1.15 (1.10, 1.21) * |
| 1 | | | 3,168 | 12,265 | 7,901 | 53,831 | 1.20 (1.14, 1.25) * |
| 2 | | | 3,158 | 12,275 | 7,839 | 53,893 | 1.21 (1.15, 1.27) * |
| 3 | | | 3,072 | 12,361 | 7,861 | 53,871 | 1.14 (1.09, 1.20) * |
| 4 | | | 3,060 | 12,373 | 7,720 | 54,012 | 1.16 (1.11, 1.22) * |
| 5 | | | 2,999 | 12,434 | 7,530 | 54,202 | 1.17 (1.12, 1.23) * |
| 6 | | | 3,004 | 12,429 | 7,622 | 54,110 | 1.14 (1.08, 1.19) * |
| 7 | | | 2,875 | 12,558 | 7,357 | 54,375 | 1.16 (1.10, 1.22) * |

^a^Adjusted for for daily mean temperature, daily mean surface precipitation amount, and daily mean relative humidity.

^* Statistically significant after Bonferroni correction for multiple comparisons at level 0.05.^

**Table S4.** Adjusted odds ratio (adjusted OR) for the association between wildfire smoke PM_2.5_ exposure and asthma exacerbation in adults, across 0- to 7-day lags, in the contiguous USA during April–August, 2006–2019, accounting for cluster standard errors.

| No. of days before exacerbation | | No. of exposed case | No. of unexposed case | No. of exposed control | No. of unexposed control | Adjusted OR^a^ (95% CIs) |
| --- | --- | --- | --- | --- | --- | --- |
| 0 | 3,521 | | 15,414 | 9,333 | 66,407 | 1.27 (1.19, 1.36) * |
| 1 | 3,621 | | 15,314 | 9,239 | 66,501 | 1.35 (1.25, 1.45) * |
| 2 | 3,615 | | 15,320 | 9,052 | 66,688 | 1.38 (1.28, 1.48) * |
| 3 | 3,511 | | 15,424 | 9,119 | 66,621 | 1.31 (1.21, 1.41) * |
| 4 | 3,514 | | 15,421 | 9,026 | 66,714 | 1.30 (1.21, 1.39) * |
| 5 | 3,449 | | 15,486 | 8,732 | 67,008 | 1.34 (1.24, 1.44) * |
| 6 | 3,416 | | 15,519 | 8,829 | 66,911 | 1.29 (1.21, 1.37) * |
| 7 | 3,310 | | 15,625 | 8,592 | 67,148 | 1.30 (1.20, 1.41) * |

^aAdjusted for daily mean temperature, daily mean surface precipitation amount, and daily mean relative humidity.^

^* Statistically significant after Bonferroni correction for multiple comparisons at level 0.05.^

**Table S5.** Adjusted odds ratio (adjusted OR) for the association between wildfire smoke PM_2.5_ exposure and asthma exacerbation in adults with the first exacerbation, across 0- to 7-day lags, in the contiguous USA during April–August, 2006–2019.

|  | No. of days before exacerbation | No. of exposed case | | No. of unexposed case | No. of exposed control | No. of unexposed control | Adjusted OR^a^ (95% CIs) |
| --- | --- | --- | --- | --- | --- | --- | --- |
| 0 | | | 949 | 5,205 | 3,418 | 21,198 | 1.15 (1.07, 1.23) * |
| 1 | | | 980 | 5,174 | 3,320 | 21,296 | 1.21 (1.13, 1.30) * |
| 2 | | | 972 | 5,182 | 3,377 | 21,239 | 1.22 (1.12, 1.32) * |
| 3 | | | 950 | 5,204 | 3,341 | 21,275 | 1.13 (1.05, 1.22) * |
| 4 | | | 963 | 5,191 | 3,362 | 21,254 | 1.18 (1.10, 1.27) * |
| 5 | | | 972 | 5,182 | 3,290 | 21,326 | 1.19 (1.11, 1.28) * |
| 6 | | | 963 | 5,191 | 3,385 | 21,231 | 1.15 (1.07, 1.23) * |
| 7 | | | 911 | 5,243 | 3,195 | 21,421 | 1.18 (1.10, 1.26) * |

^a^Adjusted for daily mean temperature, daily mean surface precipitation amount, and daily mean relative humidity.

^* Statistically significant after Bonferroni correction for multiple comparisons at level 0.05.^

**Table S6.** Adjusted odds ratio (adjusted OR) for the association between wildfire smoke PM_2.5_ exposure (per 1ug/m^3^ increase), across 0- to 7-day lags, in the contiguous USA during April–August, 2006–2019.

| No. of days before exacerbation | | Adjusted OR^a^ (95% CIs) | |  |
| --- | --- | --- | --- | --- |
|  | 0 | | 1.02 (1.01, 1.02) * | |
|  | 1 | | 1.03 (1.02, 1.03) * | |
|  | 2 | | 1.03 (1.02, 1.04) * | |
|  | 3 | | 1.02 (1.01, 1.03) * | |
|  | 4 | | 1.02 (1.01, 1.03) * | |
|  | 5 | | 1.03 (1.02, 1.04) * | |
|  | 6 | | 1.03 (1.02, 1.04) * | |
|  | 7 | | 1.03 (1.02, 1.04) * | |

^a^Adjusted for daily mean temperature, daily mean surface precipitation amount, and daily mean relative humidity.

^* Statistically significant after Bonferroni correction for multiple comparisons at level 0.05.^

**Table S7.** Adjusted odds ratio (adjusted OR) for the association between wildfire smoke PM_2.5_ exposure and asthma exacerbation in adults, across 0- to 7-day lags, adjusting for co-pollutants, in the contiguous USA during April–August, 2006–2019.

| No. of days before exacerbation | Adjusted NO_2_ | Adjusted O_3_ |
| --- | --- | --- |
|  | Adjusted OR^a^ (95% CIs) | Adjusted OR^b^ (95% CIs) |
| 0 | 1.15 (1.10, 1.20) * | 1.16 (1.11, 1.21) * |
| 1 | 1.22 (1.16, 1.28) * | 1.24 (1.19, 1.30) * |
| 2 | 1.22 (1.16, 1.27) * | 1.24 (1.19, 1.30) * |
| 3 | 1.13 (1.08, 1.19) * | 1.16 (1.11, 1.20) * |
| 4 | 1.18 (1.13, 1.24) * | 1.20 (1.15, 1.25) * |
| 5 | 1.20 (1.14, 1.25) * | 1.21 (1.16, 1.27) * |
| 6 | 1.15 (1.09, 1.20) * | 1.16 (1.10, 1.21) * |
| 7 | 1.18 (1.13, 1.23) * | 1.19 (1.14, 1.25) * |

^a^Adjusted for daily mean temperature, daily mean surface precipitation amount, and daily mean relative humidity, and daily NO_2_.

^b^Adjusted for daily mean temperature, daily mean surface precipitation amount, and daily mean relative humidity, and daily O_3_.

^* Statistically significant after Bonferroni correction for multiple comparisons at level 0.05.^

**Table S8.** Condition name and code for asthma exacerbation

| Standard concept name | SNOMED code |
| --- | --- |
| Acute severe refractory exacerbation of asthma | 733858005 |
| Acute exacerbation of asthma | 708038006 |
| Acute exacerbation of chronic obstructive airways disease with asthma | 1751000119100 |
| Exacerbation of intermittent asthma | 425969006 |
| Acute exacerbation of moderate persistent asthma | 135171000119106 |
| Acute severe exacerbation of severe persistent asthma | 707979007 |
| Acute exacerbation of allergic asthma | 708093000 |
| Exacerbation of asthma | 281239006 |
| Acute severe exacerbation of asthma | 708090002 |
| Acute exacerbation of mild persistent asthma | 135181000119109 |
| Acute severe exacerbation of immunoglobin E-mediated allergic asthma | 708095007 |
| Acute severe exacerbation of moderate persistent asthma | 707980005 |
| Exacerbation of moderate persistent asthma | 707446004 |
| Exacerbation of severe persistent asthma | 707447008 |
| Acute severe exacerbation of mild persistent asthma | 707981009 |
| Acute exacerbation of intrinsic asthma | 708094006 |
| Exacerbation of mild persistent asthma | 707445000 |
| Moderate acute exacerbation of asthma | 734905008 |
| Acute severe exacerbation of intrinsic asthma | 708096008 |
| Acute exacerbation of chronic asthmatic bronchitis | 442025000 |
| Acute exacerbation of asthma co-occurrent with allergic rhinitis | 99031000119107 |
| Chronic obstructive asthma co-occurrent with acute exacerbation of asthma | 10692721000119102 |
| Acute severe exacerbation of moderate persistent allergic asthma | 10676431000119103 |
| Exacerbation of allergic asthma due to infection | 782520007 |

SNOMED code: standardized medical diagnosis codes used in EHR data.

**Table S9.** Descriptive statistics of meteorological variables and ambient air pollutants for both cases and controls at lag days preceding asthma exacerbation events.

| Variable |  | Mean | SD | Median | Q1 | Q3 | Min | Max |
| --- | --- | --- | --- | --- | --- | --- | --- | --- |
| Temperature (K) |  |  |  |  |  |  |  |  |
| 0-day lag |  | 292.21 | 8.66 | 293.41 | 286.42 | 298.17 | 255.67 | 315.36 |
| 1-day lag |  | 292.07 | 8.73 | 293.30 | 286.22 | 298.15 | 255.42 | 315.16 |
| 2-day lag |  | 291.93 | 8.80 | 293.20 | 286.02 | 298.09 | 250.58 | 315.34 |
| 3-day lag |  | 291.79 | 8.87 | 293.05 | 285.80 | 298.00 | 252.30 | 315.33 |
| 4-day lag |  | 291.67 | 8.94 | 292.98 | 285.57 | 297.95 | 252.73 | 315.36 |
| 5-day lag |  | 291.57 | 8.99 | 292.86 | 285.38 | 297.88 | 255.16 | 315.34 |
| 6-day lag |  | 291.45 | 9.05 | 292.72 | 285.24 | 297.84 | 253.91 | 315.36 |
| 7-day lag |  | 291.28 | 9.13 | 292.55 | 284.98 | 297.75 | 255.25 | 315.36 |
| Relative Humidity (%) |  |  |  |  |  |  |  |  |
| 0-day lag |  | 69.63 | 18.71 | 74.89 | 65.17 | 81.56 | 4.81 | 97.64 |
| 1-day lag |  | 69.45 | 18.72 | 74.53 | 64.88 | 81.45 | 4.29 | 98.07 |
| 2-day lag |  | 69.49 | 18.71 | 74.60 | 64.85 | 81.49 | 3.90 | 97.64 |
| 3-day lag |  | 69.55 | 18.76 | 74.74 | 64.78 | 81.65 | 4.26 | 97.64 |
| 4-day lag |  | 69.48 | 18.79 | 74.71 | 64.76 | 81.56 | 4.29 | 97.64 |
| 5-day lag |  | 69.55 | 18.78 | 74.82 | 64.96 | 81.57 | 3.90 | 97.64 |
| 6-day lag |  | 69.62 | 18.80 | 74.94 | 65.01 | 81.61 | 4.26 | 99.38 |
| 7-day lag |  | 69.55 | 18.82 | 74.85 | 64.83 | 81.68 | 4.29 | 97.64 |
| Surface precipitation amount (kg/m^2^) |  |  |  |  |  |  |  |  |
| 0-day lag |  | 2.95 | 6.59 | 0.12 | 0.00 | 2.55 | 0.00 | 114.87 |
| 1-day lag |  | 2.90 | 6.53 | 0.11 | 0.00 | 2.40 | 0.00 | 107.26 |
| 2-day lag |  | 2.82 | 6.43 | 0.11 | 0.00 | 2.32 | 0.00 | 111.84 |
| 3-day lag |  | 2.87 | 6.58 | 0.11 | 0.00 | 2.37 | 0.00 | 159.01 |
| 4-day lag |  | 2.88 | 6.53 | 0.11 | 0.00 | 2.40 | 0.00 | 123.09 |
| 5-day lag |  | 2.90 | 6.56 | 0.11 | 0.00 | 2.40 | 0.00 | 111.84 |
| 6-day lag |  | 2.83 | 6.44 | 0.11 | 0.00 | 2.34 | 0.00 | 93.46 |
| 7-day lag |  | 2.92 | 6.63 | 0.11 | 0.00 | 2.40 | 0.00 | 114.87 |
| Wildfire smoke PM_2.5_ (ug/m^3^) |  |  |  |  |  |  |  |  |
| 0-day lag |  | 0.43 | 1.72 | 0.00 | 0.00 | 0.00 | 0.00 | 55.09 |
| 1-day lag |  | 0.42 | 1.68 | 0.00 | 0.00 | 0.00 | 0.00 | 55.09 |
| 2-day lag |  | 0.44 | 1.74 | 0.00 | 0.00 | 0.00 | 0.00 | 55.09 |
| 3-day lag |  | 0.43 | 1.71 | 0.00 | 0.00 | 0.00 | 0.00 | 55.09 |
| 4-day lag |  | 0.41 | 1.63 | 0.00 | 0.00 | 0.00 | 0.00 | 55.09 |
| 5-day lag |  | 0.40 | 1.62 | 0.00 | 0.00 | 0.00 | 0.00 | 55.09 |
| 6-day lag |  | 0.39 | 1.59 | 0.00 | 0.00 | 0.00 | 0.00 | 55.09 |
| 7-day lag |  | 0.39 | 1.67 | 0.00 | 0.00 | 0.00 | 0.00 | 55.09 |
| NO_2_ (ppb) |  |  |  |  |  |  |  |  |
| 0-day lag |  | 6.28 | 5.50 | 4.77 | 2.23 | 8.49 | 0.10 | 57.31 |
| 1-day lag |  | 6.12 | 5.42 | 4.63 | 2.17 | 8.22 | 0.08 | 56.40 |
| 2-day lag |  | 6.00 | 5.36 | 4.49 | 2.12 | 8.13 | 0.10 | 56.40 |
| 3-day lag |  | 5.97 | 5.31 | 4.47 | 2.11 | 8.12 | 0.12 | 56.40 |
| 4-day lag |  | 5.95 | 5.27 | 4.44 | 2.11 | 8.08 | 0.13 | 56.40 |
| 5-day lag |  | 6.02 | 5.33 | 4.51 | 2.13 | 8.12 | 0.08 | 57.31 |
| 6-day lag |  | 6.19 | 5.44 | 4.66 | 2.19 | 8.37 | 0.12 | 56.40 |
| 7-day lag |  | 6.30 | 5.54 | 4.76 | 2.24 | 8.55 | 0.10 | 57.31 |
| O_3_ (ppb) |  |  |  |  |  |  |  |  |
| 0-day lag |  | 31.13 | 6.63 | 31.31 | 26.86 | 35.38 | 1.67 | 66.07 |
| 1-day lag |  | 31.21 | 6.58 | 31.35 | 26.99 | 35.39 | 1.78 | 68.59 |
| 2-day lag |  | 31.29 | 6.57 | 31.38 | 27.09 | 35.46 | 2.78 | 64.29 |
| 3-day lag |  | 31.26 | 6.56 | 31.38 | 27.06 | 35.39 | 1.42 | 64.29 |
| 4-day lag |  | 31.28 | 6.53 | 31.40 | 27.11 | 35.42 | 1.28 | 64.66 |
| 5-day lag |  | 31.23 | 6.53 | 31.37 | 27.07 | 35.38 | 1.78 | 64.29 |
| 6-day lag |  | 31.16 | 6.59 | 31.30 | 26.98 | 35.35 | 1.67 | 64.29 |
| 7-day lag |  | 31.03 | 6.59 | 31.26 | 26.84 | 35.21 | 1.67 | 66.07 |

SD: Standard deviation.

K: Kelvin

ppb: Parts per billion

**Table S10.** Distribution of 3-Digit ZIP Code Land Area by U.S. Region.

| **Region** | **States Included** | **Minimum 3-Digit ZIP Area (km^2^)** | **Median 3-Digit ZIP Area (×10^4^ km^2^)** | **Maximum 3-Digit ZIP Area (×10^4^ km^2^)** |
| --- | --- | --- | --- | --- |
| Midwest | ND, SD, NE, KS, MN, IA, MO, WI, IL, MI, IN, OH | 18.49 | 0.69 | 4.43 |
| Northeast | ME, VT, NH, MA, RI, CT, NY, PA, NJ, DE, DC, MD | 0.16 | 0.15 | 1.44 |
| Southeast | KY, WV, VA, TN, NC, SC, GA, FL, AL, MS, AR, LA | 67.60 | 0.57 | 2.22 |
| Southwest | AZ, NM, OK, TX | 1.19 | 1.29 | 6.23 |
| West | WA, OR, CA, ID, MT, WY, NV, UT, CO | 0.49 | 1.13 | 9.68 |

Fig. S1 Five USA state regions for stratified analysis.

Fig. S2 Adjusted odds ratio (adjusted OR) for the association between wildfire smoke PM_2.5_ exposure and asthma exacerbation in adults, across 0- to 7-day lags, in the contiguous USA during April–August, 2006–2019; stratified by year.

(a)

(b)

Fig. S3. Spatial distribution of wildfire smoke PM_2.5_ exposure by 3-digit ZIP code (ZIP3) region in the contiguous USA during April–August, 2006–2019. (a) Percentage of days with wildfire PM_2.5_ > 0 µg/m³. (b) Mean wildfire PM_2.5_ concentration (µg/m³) among days with PM_2.5_ > 0.
